# OSTRFPD: Multifunctional Tool for Genome-Wide Short Tandem Repeat Analysis for DNA, Transcripts, and Amino Acid Sequences with Integrated Primer Designer

**DOI:** 10.1177/1176934319843130

**Published:** 2019-04-23

**Authors:** Vivek Bhakta Mathema, Arjen M Dondorp, Mallika Imwong

**Affiliations:** 1 Department of Molecular Tropical Medicine and Genetics, Faculty of Tropical Medicine, Mahidol University, Bangkok, Thailand; 2 Mahidol-Oxford Tropical Medicine Research unit, Faculty of Tropical Medicine, Mahidol University, Bangkok, Thailand; 3 Centre for Tropical Medicine, Churchill Hospital, Oxford, UK

**Keywords:** microsatellites, tandem repeat, *in silico* mining, flanking sequence, genotyping marker

## Abstract

Microsatellite mining is a common outcome of the *in silico* approach to genomic studies. The resulting short tandemly repeated DNA could be used as molecular markers for studying polymorphism, genotyping and forensics. The omni short tandem repeat finder and primer designer (OSTRFPD) is among the few versatile, platform-independent open-source tools written in Python that enables researchers to identify and analyse genome-wide short tandem repeats in both nucleic acids and protein sequences. OSTRFPD is designed to run either in a user-friendly fully featured graphical interface or in a command line interface mode for advanced users. OSTRFPD can detect both perfect and imperfect repeats of low complexity with customisable scores. Moreover, the software has built-in architecture to simultaneously filter selection of flanking regions in DNA and generate microsatellite-targeted primers implementing the Primer3 platform. The software has built-in motif-sequence generator engines and an additional option to use the dictionary mode for custom motif searches. The software generates search results including general statistics containing motif categorisation, repeat frequencies, densities, coverage, guanine–cytosine (GC) content, and simple text-based imperfect alignment visualisation. Thus, OSTRFPD presents users with a quick single-step solution package to assist development of microsatellite markers and categorise tandemly repeated amino acids in proteome databases. Practical implementation of OSTRFPD was demonstrated using publicly available whole-genome sequences of selected *Plasmodium* species. OSTRFPD is freely available and open-sourced for improvement and user-specific adaptation.

## Introduction

During the past decade, there has been rapid advancement in whole-genome sequencing (WGS) technologies, transcriptomes and proteomics. Most of these high-throughput sequencing platforms generate large data sets, which are often made publicly available as online repositories.^1^ An *in silico* approach can use these resources to investigate different features of an organism including phylogeny, genotyping, and mutation. Repetitive elements, in particular, short tandem repeats (STRs) of DNA are characteristic features of eukaryotes. Microsatellites are the most common form of STRs, typically with motif lengths of 1 to 6 bp, and can occur with copy numbers ranging from 5 to more than 100, depending upon the motif type and organism. These repeats are often interrupted by random insertions or deletions of nucleotides to form an imperfect repeat.^2,3^ Gene isolation and primer design of multiple eukaryotic pathogens, such as *Plasmodium*, are particularly challenging owing to the presence of large numbers of microsatellites. For example, *Plasmodium falciparum* 3D7 has an extremely AT-rich genome (80.16%) and is known to harbour microsatellites, which constitute 6% to 7% of its entire genome.^4^ This repetitive DNA may contribute to diversity and even virulence of the pathogen as microsatellite instability in coding regions is more likely to produce mutant proteins.^5^ The effects of such repeats in coding regions are also reflected in subsequent transcripts and ribosomal RNAs. Moreover, in-depth analysis of protein repeats is increasing owing to their role in the structure, function, evolution, and host–parasite interactions of eukaryotes.^6–8^ Thus, an integrated **in silico** approach, combining genome-wide microsatellite searches with the ability to directly identify repetitive RNA and amino acid sequences, would provide deeper insights into the overall distribution of repetitive sequences in an organism. Establishment of online repertories, such as PlasmoDB^9,10^ and other WGS projects entirely dedicated to a single organism with an integrated platform providing genome, transcriptome, and proteome data, has remained vital for such studies.^11–14^ Usage of these resources with a comprehensive *in silico* approach would greatly enhance effectiveness and reduce the time and cost of research projects. Publishers of major scientific journals, such as BioMed Central and PLOS, are encouraging publication of wide-range open-source bioinformatics tools, which will benefit the entire scientific community.^15–17^ The omni short tandem repeat finder and primer designer (OSTRFPD) has been designed to address some of these key issues by providing a simple yet useful tool to rapidly identify and categorise repetitive nucleic or amino acid sequences and to assist in the development of microsatellite-targeted primers with minimum user input and programming knowledge.

### Implementation

**Figure 1.**
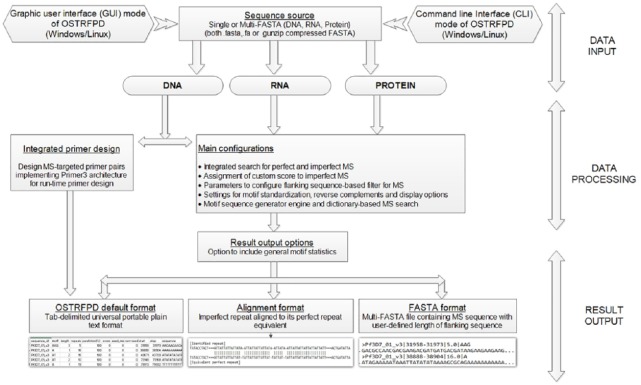
Schematics of OSTRFPD software architecture and workflow. OSTRFPD can either be used as command line console with arguments or as a fully featured graphic user interface tool. Single or multi-FASTA file (eg, .fasta, .fa, and .gz ‘gunzip-compressed fasta’) for nucleic acid or protein is directly accepted as data source. All type of sequences can be scanned for short tandem repeats and primers can be simultaneously designed for DNA-associated microsatellites using built-in flanking sequence filter and primer3 plugin. Results can be generated with the option to include general statistics report. Results generated can be of 3 major types: (1) ‘Default’ with tab-delimited values and associated headers (2) ‘Alignment’ or ‘Imperfect Alignment only’ format with alignments of repeats for both perfect and imperfect repeat, and (3) ‘FASTA’ as portable multi-FASTA format containing target microsatellite with flanking sequences. MS indicates microsatellites; OSTRFPD, omni short tandem repeat finder and primer designer.

OSTRFPD has been designed for molecular researchers with little or no computer programming background in mind and optimised for small- (approximately 5 Kbp) to medium-sized (approximately 50 Mbp) FASTA sequences. The architecture and workflow of OSTRFPD (Figure 1) consist mainly of FASTA sequences (DNA, RNA, or proteins), which are scanned for user-configurable repetitive units. The software supports detection of both perfect and imperfect repeats with low complexity, which widens the range of potential STR analyses. Configuration options for results can vary based on sequence type and the anticipated output format. The format of the output can be tabulated values (default), FASTA sequences, or alignment type. OSTRFPD has the option to display imperfect repeats in plain text alignment, comparing the imperfect sequence with its nearest perfect equivalent for visually identifying indels, gaps, and mismatches. The alignment mode also generates additional information, such as the default local alignment scores, custom scores, and a rudimentary consensus sequence, based on perfectness of the repeat. For DNA sequences, the software uses the well-established Primer3 platform with configurable parameters for simultaneously designing primers on microsatellite detection. Moreover, assuming that the primer-tag option is selected, OSTRFPD appends a user-defined tag to the 5′-tail of primers, which simplifies the process for ordering tagged primers. The dictionary-based motif search is a unique feature of OSTRFPD. The dictionary is essentially a plain text file with each custom motif listed on a new line. The dictionary must contain only 1 type of molecule (not a mixture of DNA, RNA, or proteins). During the runtime, motifs are processed automatically to filter out any duplicates or equivalent cyclic motifs. The current version of OSTRFPD only supports fixed-length motifs and single minimum repeat number-based searches, although a single dictionary file may contain collections of variable-length motifs. The dictionary mode exclusively allows searches of motifs of 1 to 30 bp or amino acids, which may enable researchers to identify user-defined simple oligonucleotides, transcription factor binding regions, or signalling peptide sequences. Dictionaries optimised for nucleotide and amino acid motifs commonly observed in *Plasmodium* species have been bundled with the OSTRFPD distribution.

## Materials and Methods

### Selection of databases

The usability of OSTRFPD was demonstrated with freely available standard reference genomic and protein databases of selected *Plasmodium* species from the PlasmoDB web server (http://plasmodb.org/common/downloads/release-36/). The sequences for whole-genome and annotated proteins were analysed for perfect repeats in *P falciparum* 3D7, *Plasmodium vivax* SAL-1, and *Plasmodium ovale curtisi* GH01. The ribosomal RNA (rRNA) sequences for *P falciparum* Dd2 28S rRNA (URS0000A6B25D), and *Plasmodium knowlesi* strain H 28S rRNA (URS0000857690) were obtained from EMBL-EMB’s RNAcentral database (https://rnacentral.org). Tandem repeats, with motif lengths of 1 to 6 bp for DNA and 1 to 2 amino acid residues for proteins, were searched using MISA-formatted strings with default minimum repeat settings of ‘14,7,5,4,4,4’ and ‘7,5’ for DNA (Supplemental Figure 1) and protein sequences (Supplemental Figure 2), respectively. Equivalent command line parameters for DNA and protein sequences are ‘python3 ostrfpd.py -scan dna -input source_rna_fasta -unitmin 1 -unitmax 3 -misa 12,6,4’ and ‘python3 ostrfpd.py -scan protein -input source_protein_fasta -unitmin 1 -unitmax 2 -misa 7,5’, respectively. Similarly, an RNA scan was conducted for motifs of 1 to 3 bp using the MISA-formatted string ‘12,6,4’ for searching minimum repeats of 12, 6, and 4, respectively (Supplemental Figure 3). Equivalent command line parameters are ‘python3 ostrfpd.py -scan rna -input source_rna_fasta -unitmin 1 -unitmax 3 -misa 12,6,4’. For primer design, in addition to the default settings, the fixed motif length, minimum repeats, and maximum Poly-X’s were set to 3, 8, and 3, respectively (Supplemental Figure 4). The flanking sequence filter with parameter ‘1,5’ was applied to minimise generation of undesirable primers with low-numbered repeats in flanking regions. Equivalent command line parameters are ‘python3 ostrfpd.py -scan dna -input source_fasta -fsc 1,5 -unitmin 3 -unitmax 3 -min 8 -fix true -primer true’.

### Software prerequisites for running OSTRFPD

OSTRFPD is freely available under the GNU General Public License (GPL) (https://www.gnu.org/licences/gpl-3.0.en.html). The software was tested for proper operation in both Windows (version 7, 10) and Linux Ubuntu (version 16.04), provided that at least Python 3.5, PyQt5 5.9.1, and Biopython 1.7 are correctly installed.^18,19^ The software uses Python’s built-in powerful regular expression engine to identify patterns within DNA, RNA, or amino acid sequences and locate STRs. To generate primers, users can either directly implement standalone primer3 binaries supplied with the software package or individually compile primers from the official source (https://sourceforge.net/projects/primer3/files/primer3/1.1.4/). The details of each parameter for primer design can be obtained from primer3 documentation (http://primer3.sourceforge.net/primer3_manual.htm).^20^

### Ease of operation

**Figure 2.**
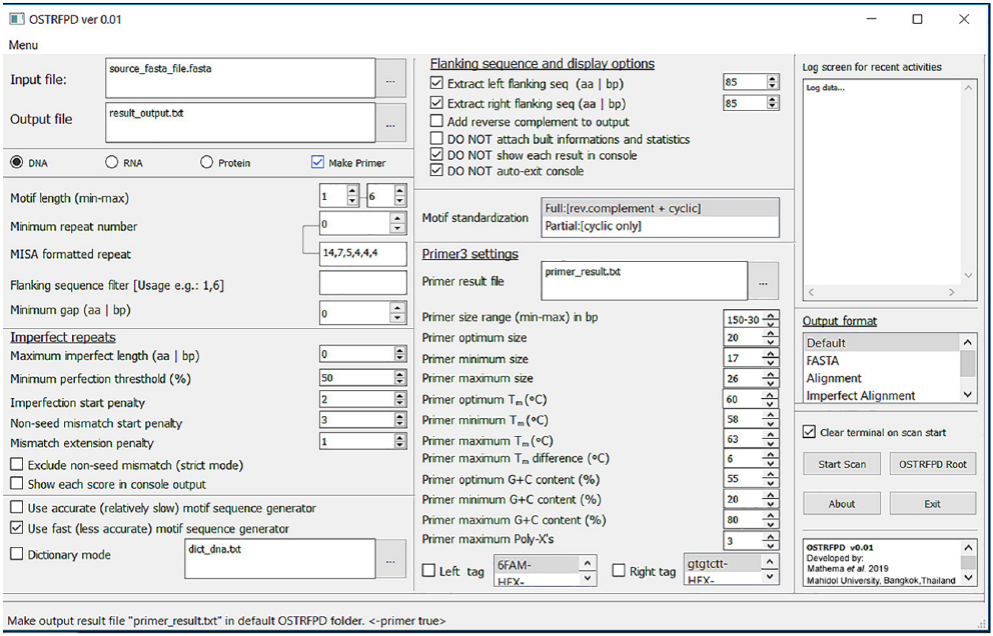
Simplified graphical user interface (GUI) for data input. OSTRFPD provides a user-friendly graphical interface which can be initialised using simple argument ‘python3 ostrfpd.py -gui true’ in console or terminal. The user interface has decent level of built-in error handling modules to minimise invalid data input. Graphical user interface works along with display of console screen. Simple tooltip displayed on status bar provides a short description of each option under consideration and shows example of command line interface parameters whenever feasible as ‘<eg, -command value>’. OSTRFPD indicates omni short tandem repeat finder and primer designer.

**Figure 3.**
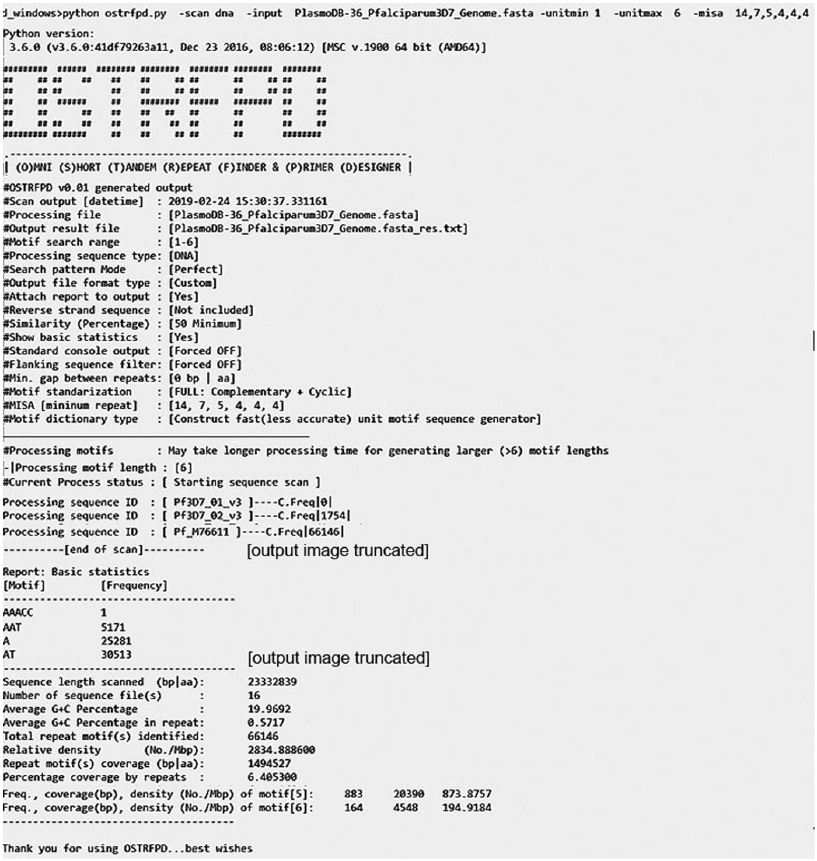
Demonstration of a command line interface (CLI) data input. OSTRFPD has an advance option for CLI that can be initialised using no argument ‘python3 ostrfpd.py’ or ‘python3 ostrfpd.py -gui false’ in console or terminal. The CLI mode allows to use OSTRFPD for batch operation as well as a plugin script that can be implemented by other software. Representative images are truncated to save space. OSTRFPD indicates omni short tandem repeat finder and primer designer.

OSTRFPD can either run as fully featured standalone OS-specific binaries or run directly from the source code within a platform-independent Python environment. OSTRFPD supports fully featured graphical user interface (GUI) or command line interface (CLI) in a Windows console or Linux terminal. The GUI mode (Figure 2) is equipped with tool tips and basic level of error handling modules to avoid invalid or unintentional inputs. A typical GUI mode can be initiated using parameters ‘python3 ostrfpd.py -gui true’ in the console or terminal. The CLI mode (Figure 3) is suitable for advanced users who choose to conduct batch operations or implement OSTRFPD as a plugin for their own utilities. Command line interface mode is activated by default. The software generates user-configurable detailed output that can be retrieved as a tab-delimited report file (default), FASTA sequences, or in an alignments format. The details of each parameter and the syntax in the CLI mode can be accessed by following software documentation or using the built-in help ‘—help’ argument.

## Results

### Practical implementation of OSTRFPD

**Figure 4.**
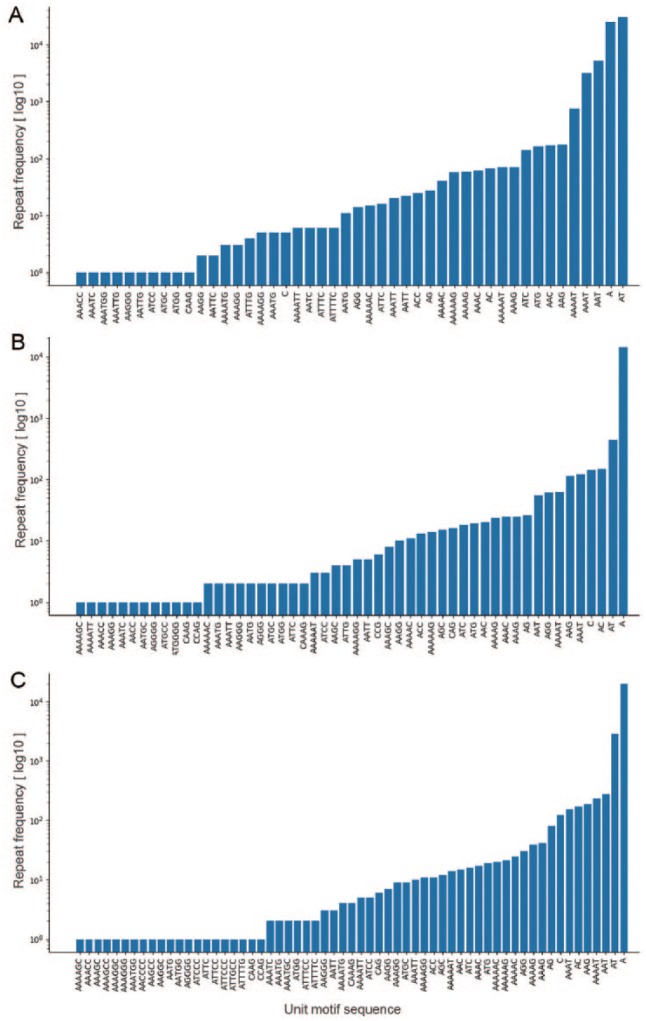
Frequency distribution of unit microsatellite repeats in *Plasmodium* species using OSTRFPD. Entire genome sequences of (A) *Plasmodium falciparum* 3D7, (B) *Plasmodium vivax* SAL-1, and (C) *Plasmodium ovale curtisi* were searched for 1 to 6 bp unit motif with minimum repeat number of 14,7,5,4,4, and 4, respectively. Search criteria were limited to maximum 6 nucleotide long motif due to large number of patterns involved. Each letters in x-axis represents their regular notation for DNA nucleotide residues. Equivalent command line parameters were supplied as ‘python3 ostrfpd.py -scan dna -input source_nucleotide_fasta -unitmin 1 -unitmax 6 -misa 14,7,5,4,4,4’. OSTRFPD indicates omni short tandem repeat finder and primer designer.

**Figure 5.**
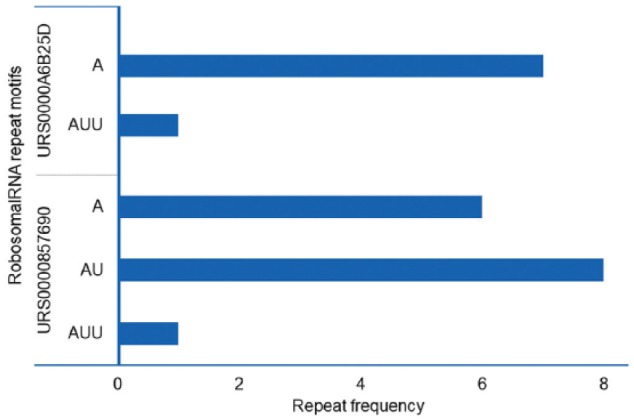
Frequency distribution of unit RNA repeat motif in rRNA using OSTRFPD. The individual rRNA sequences for (A) *Plasmodium falciparum* Dd2 28S rRNA (URS0000A6B25D) and (B) *Plasmodium knowlesi* strain H 28S rRNA (URS0000857690) were directly scanned for tandem repeats. The sequences were reached for 1 to 3 bp unit motif with minimum repeat number of 12, 6, and 4, respectively. Equivalent command line parameters were supplied as ‘python3 ostrfpd.py -scan rna -input ./rna_seq/source_rna_fasta -unitmin 1 -unitmax 3 -misa 12,6,4’. OSTRFPD indicates omni short tandem repeat finder and primer designer; rRNA, ribosomal RNA.

**Figure 6.**
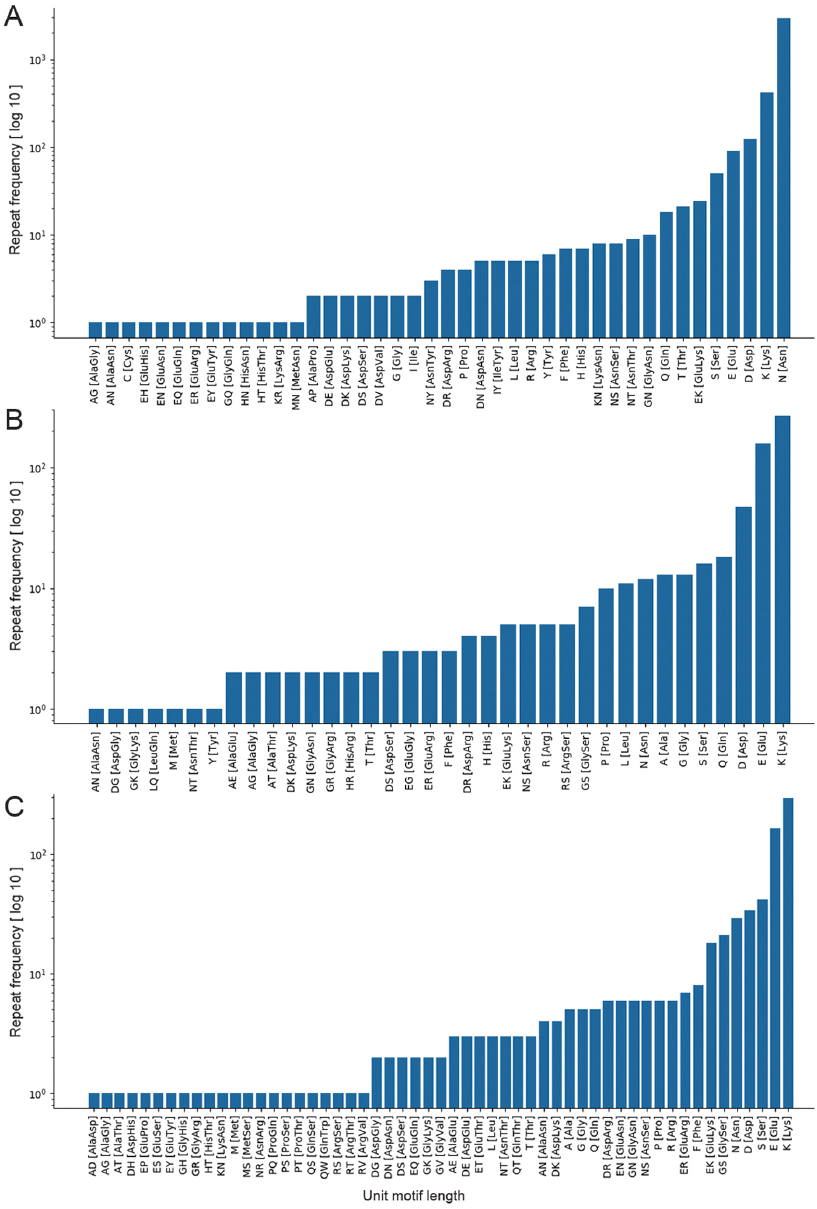
Frequency distribution of unit amino acid repeat motifs in *Plasmodium* species using OSTRFPD. Entire known protein sequences of (A) *Plasmodium falciparum* 3D7, (B) *Plasmodium vivax* SAL-1, and (C) *Plasmodium ovale curtisi*. GH01 were searched for 1 to 2 amino acid unit motif with minimum repeat number of 7 and 5, respectively. Search criteria for the representative graph was limited to maximum of 2 amino acid unit motifs due to large number of unique motif type involved. Each letters in x-axis represents regular notation for amino acid residues. Equivalent command line parameters were supplied as ‘python3 ostrfpd.py -scan protein -input source_protein_fasta -unitmin 1 -unitmax 2 -misa 7,5’.

As an example, the microsatellite (Table 1) and amino acid residues (Table 2) identified during the demonstration reflect characteristic features of the extremely AT-rich *Plasmodium* genome.^4,21^ The *P falciparum* genome had the highest number of microsatellites (66 146) with an average density of 2835 microsatellites/million base pair (Mbp), and the total number of tandemly repeated amino acid residues was 3803. In addition, A, AT, and AAT were among the most frequently repeated motifs, comprising more than 50% of the total motifs in each *Plasmodium* species. OSTRFPD can be configured to automatically generate computationally feasible primers targeting such microsatellite motifs. Process of primer design begins with identification of microsatellite, subsequent analysis of its flanking sequences, and selection of computationally feasible primer pair that can amplify the region containing tandem repeats (Supplemental Figure 5). Microsatellite-targeted candidate genotyping primers were designed for the relatively less studied *P ovale curtisi* GH01 (Supplemental Table 1). For amino acid repeats, the highest number was detected in *P falciparum* (3803) with an average density of 908 repeats per million residues (Table 2). In addition, each motif-sequence and the associated frequency distribution of microsatellites (Figure 4), rRNA repeat motifs (Figure 5), and amino acid sequences (Figure 6) were automatically categorised to clearly elucidate the types of repeats involved.

**Table 1.** Detection of microsatellite using OSTRFPD in different species of *Plasmodium*.

Features	*Plasmodium falciparum* 3D7	*Plasmodium vivax* SAL-1	*Plasmodium ovale curtisi* GH01
Microsatellite loci	66 146	15 787	24 420
% Coverage of genome	6.41	1.13	1.56
Average density (loci/Mbp)	2834.88	584.40	729.40
Average % GC content of sequence	19.97	32.04	22.12
Average % GC content of microsatellite	0.57	2.59	1.60

Abbreviations: OSTRFPD, omni short tandem repeat finder and primer designer; GC, guanine–cytosine.

Summary of microsatellite obtained by conducting genome-wide search for 1 to 6 base pair (bp) unit motif using default settings with minimum repeat settings of 14,7,5,4,4, and 4, respectively. Equivalent command line parameters were supplied as ‘python3 ostrfpd.py -scan dna -input source_nucleotide_fasta -unitmin 1 -unitmax 6 -misa 14,7,5,4,4,4’.

**Table 2.** Detection of amino acid repeats using OSTRFPD in different species of *Plasmodium*.

Features	*Plasmodium falciparum* 3D7	*Plasmodium vivax* SAL-1	*Plasmodium ovale curtisi* GH01
Amino acid repeat loci	3803	640	733
% Coverage of proteome	0.93	0.15	0.17
Average density (loci/10^6^ aa)	908.24	163.30	165.98

Abbreviation: OSTRFPD, omni short tandem repeat finder and primer designer.

Summary of amino acid repeat conducted for proteome-wide search for 1 to 2 amino acid (aa) unit motif repeat using default settings with minimum repeats of 7 and 5, respectively. Equivalent command line parameters were supplied as ‘python3 ostrfpd.py -scan protein -input source_protein_fasta -unitmin 1 -unitmax 2 -misa 7,5’.

### Identification and simple alignment view of imperfect repeats

**Figure 7.**
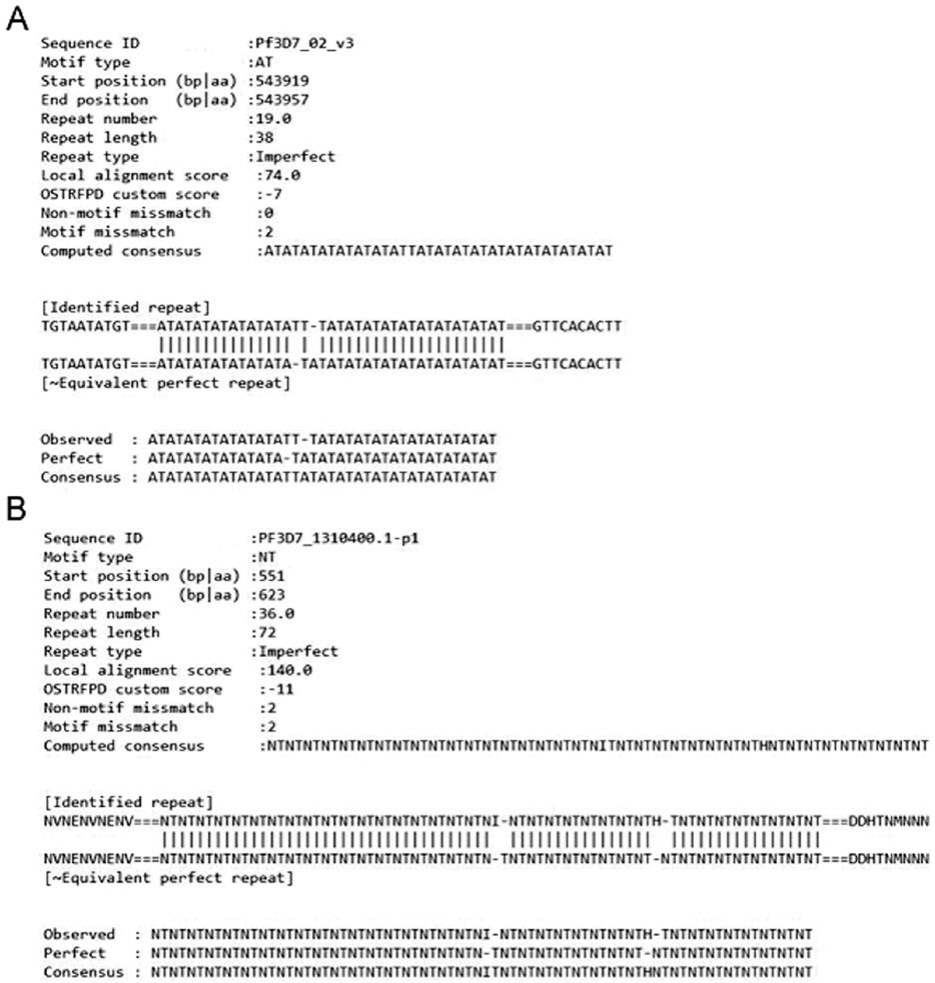
Visualisation of imperfect repeat using simple text-based alignment. The imperfect short tandem repeats for randomly picked (A) DNA and (B) protein sequences of *Plasmodium falciparum* 3D7 were visualised as simple text-based alignment comparing each imperfect repeat with their near equivalent perfect repeat. Results also show other parameters including Biopythons’ default local alignment score, repeat number, and frequency of non-motif indels within the imperfect repeats. Letters indicate standard representation of nucleotides and amino acids. Equivalent command line parameters for obtaining similar samples of imperfect alignment can be supplied for (A) DNA as ‘python3 ostrfpd.py -scan dna -input source_dna_fasta -unitmin 1 -unitmax 6 -imperfect 10 -imalign true’ and (B) protein as ‘python3 ostrfpd.py -scan protein -input source_protein_fasta -unitmin 1 -unitmax 3 -imperfect 10 -imalign true’.

An in-depth analysis of imperfect microsatellites could be conducted by visualising the simple text-based alignment to identify indels. The example provided illustrates the results displayed for an imperfect alignment of a randomly selected *Plasmodium* DNA (Figure 7A) and protein (Figure 7B) sequence with their closest corresponding equivalent perfect repeats. In addition, the result displays Biopython’s default local alignment scores, non-motif indels, and custom scores along with other minor parameters by default (Figure 7). Similar results can be obtained with user-specified command line parameters for DNA: ‘python3 ostrfpd.py -scan dna -input source_dna_fasta -unitmin 1 -unitmax 3 -imperfect 10 -imalign true’ and for protein: ‘python3 ostrfpd.py -scan protein -input source_protein_fasta -unitmin 1 -unitmax 3 -imperfect 10 -imalign true’.

### Processing speed, CPU, and memory usage

On average, the speed of sequence searches for perfect repeats of 1 to 6 bp long DNA motifs in ‘fast search’ mode is approximately 200 seconds for nearly 30 Mbp of sequence with a 2.4 GHz Core i5 processor containing 4 GB DDR3 RAM and 3 Mb cache memory. The search time was reduced to approximately 90 seconds for 1 to 4 bp DNA motifs under similar conditions. In contrast, for amino acid sequences totalling approximately 4 million residues, the speed of sequence searches for 1 to 3 and 1 to 2 amino acid long repeats in ‘fast search’ mode was approximately 468 and 75 seconds, respectively. However, the estimates were found to vary 5% to 10% depending on the background computing load of the system. During each scanning process, the overall CPU usage by OSTRFPD remained in the range of 15% to 35%, allowing the computer to remain operable for regular multitasking.

### Feature comparison with other microsatellite software

An overview of OSTRFPD in comparison with other common microsatellite search tools belonging to a similar category was conducted. OSTRFPD was the only software with an option to filter out microsatellite-targeted primers based on short repeats found within flanking sequences (Table 3). In addition, OSTRFPD has the unique feature of direct analysis of nucleic acid (DNA and RNA) and amino acid sequences for tandem repeats. Other than Msatcommander,^22^ OSTRFPD was the only offline tool that could simultaneously generate microsatellite-targeted primers without the need of any additional PERL scripts or manual steps (Table 3). Moreover, OSTRFPD had additional improvements over Msatcommander by identifying and categorising STRs with longer motifs. In contrast with MISA-Web^23^ and SciRoKo,^24^ OSTRFPD allowed a wider range of motif selection with the provision of filtering STRs based on multiple parameters including perfection threshold, flanking regions, and custom motifs. The dictionary-based search mode was exclusive to OSTRFPD among the other tools, which allowed precise control over motif sequences being scanned with longer motif ranges (1-30 bp) for both nucleotide and protein sequences. OSTRFPD could selectively generate alignment-formatted output for imperfect repeats with custom scores, a feature minimally available in other software.

**Table 3.** Comparison of features among different tandem repeat search tools.

Utility tool	Offline/Web	Integrated primer design^a^	Unit motif length supported^b^			Flanking sequence analysis	Custom scoring	Custom motif search^c^	Alignment view
			DNA	RNA	Proteins				
OSTRFPD	Offline	Yes	⩽10 bp	⩽10 bp	⩽4 aa	Yes	Yes	Yes	Yes
Phobos^25^	Offline	No	⩽10 kb	No	No	No	Yes	No	No
Msatcommander^22^	Offline	Yes	⩽6 bp	No	No	No	No	No	Yes
SciRoKo^24^	Offline	No	⩽6 bp	No	No	No	Yes	No	No
TRF^26^	Offline	No	⩽500 bp	No	No	Yes	Yes	No	No
WebSat^27^	Online	No	⩽6 bp	No	No	No	No	No	Yes
SSRIT^28^	Online	No	⩽10 bp	No	No	No	No	No	No
MISA-Web^23^	Online	No	⩽6 bp	No	No	No	No	No	No

Abbreviations: OSTRFPD, omni short tandem repeat finder and primer designer; TRF, tandem repeat finder.

a Ability to design and simultaneously produce primers using Primer3 without the need of additional post-processing with PERL scripts or further manual steps.

b The maximum unit motif length of tandemly repeated nucleotide or amino acid residue supported by each software.

c For OSTRFPD using dictionary-based custom motif search, the maximum length for unit motif is 30 base pair (bp) or amino acid (aa).

## Discussion

OSTRFPD provides an integrated solution for identification of perfect or imperfect STRs with low complexity and microsatellite-targeted primer design. The ease of operation and the open-source and cross-platform compatibility of the software make it a useful tool for genome- or proteome-wide surveys of small- to medium-sized sequence databases. *Plasmodium* species were suitable for validation of the STR mining capacity of this software because of their high microsatellite content and diversity.^4^ The capabilities and features of OSTRFPD for identification and categorisation of nucleic or amino acids in *Plasmodium* species suggest the ease of operations and suitable improvement over existing software.^22,23^ Other than perfect microsatellites, STRs have various forms and complexities.^29,30^ OSTRFPD partly addresses these issues by being able to detect imperfect repeats with low complexity. Specifically, the STRs that satisfy the minimum selection criteria are further examined for interruption within the bound of user-supplied imperfection limits. Moreover, these imperfect repeats can be scored and filtered based on percentage of perfectness, type of indels causing the imperfection, or the combination of both. The scoring scheme is essentially a numerical designation for the number of imperfect indels and imperfection-associated penalties that the user assigns for imperfect repetitive sequences. Similarly, perfectness is the percentage of motifs within the imperfect repeat. For example, a perfect repeat containing 10 motifs scores 100% perfectness, whereas as an imperfect repeat of the same length and motif but containing only 9 units of perfect repeats scores 90% perfectness. The ability of OSTRFPD to identify, score and present imperfect STRs, and provide output in both regular and alignment formats can foster deeper understanding of repetitive elements in genomes and proteomes.

One important bottleneck in the study of STRs is the categorisation of motifs, which may occur in cyclic, palindromic, or complimentary forms. For example, ATA_n_, AAT_n_, and TAA_n_ are cyclic equivalents of each other and thus are categorised as the same motif under partial standardisation. Full standardisation incorporates cyclic equivalents and their reverse complements under the same category of repetitive sequence. Thus, ATA_n_, AAT_n_, TAA_n_, TAT_n_, ATT_n_, and TTA_n_ will be categorised as the same motif under full standardisation. Options for both full and partial standardisation are available for nucleic acids, whereas the amino acid sequences are restricted to partial standardisation. Thus, OSTRFPD resolves this motif categorisation issue, which benefits the user by allowing the customisation of results based on the motif-sequence and the anticipated output format. Another common problem faced during microsatellite-based primer design is the occurrence of low-numbered repeats in flanking regions. For example, the occurrence of A_n_, AT_n_ within flanking regions, where n is generally less than half the value of the corresponding microsatellite detection threshold, creates problems in primer design. Manual inspection to mitigate these issues in a large data set is not often a feasible solution. The presence of a configurable scanner to filter out microsatellites flanked by sequences harbouring low-numbered repeats significantly improves optimised primer design. The implementation of all these filters to amino acid sequences is a novel feature of OSTRFPD and benefits users who wish to investigate STRs in a proteome database. Although there are several tandem repeat identification software, such as SciRoKo, Msatcommander, Phobos,^25^ TRF^26^, SSRIT,^28^ and MISA-Web, many are either closed-source or limited to detection of DNA sequences with no option for simultaneous primer design.^31^ Unlike most microsatellite tools, the ability of OSTRFPD to directly implement Primer3 without additional PERL scripts drastically reduces manual post-processing steps for the construction of microsatellite-targeted primers. A typical microsatellite motif for genotyping markers is 2 to 5 bp in length, which can be handled easily by OSTRFPD. In addition, the software provides the option to detect tandemly repeated RNA sequences, which are rarely investigated, but still might be useful for specific tasks such as ribosomal RNA, transcriptomes, and RNA virus genome analysis.^32^ These RNA-associated tandem repeats may influence protein folding, ribosomal constructs, and binding activities of their target proteins or enzymes.^33,34^ Implementation of OSTRFPD to directly evaluate tandemly repeated RNA sequences may contribute to the scant information available on studies of repetitive RNA sequences. In addition, lysine-rich STRs have been observed in different protozoal parasites, including *Plasmodium falciparum* and *Leishmania* major. These parasites may generate these STRs *de novo* to modulate host protein targeting efficiency.^8,35^ Simple amino acid repeats may provide flexibility for optimal folding of structural or functional domains; thus, the OSTRFPD may assist researchers interested in proteome-wide quantification of such repeats. Furthermore, inclusion of an option to implement a user-specified motif dictionary enables highly customisable searches for organism-specific motif identification as well as estimation of specific oligonucleotide or peptide sequence density. OSTRFPD runs relatively slower than native C-compiled tools (ie, Phobos and SciRoKo) owing to the limitation of Python’s architecture; however, the flexibility, unique features, ease of operation, and open-source nature of this software may compensate for its few drawbacks depending on the requirements of the user.

## Supplemental Material

Supplementary_Figure1_revised_xyz152315147f517 – Supplemental material for OSTRFPD: Multifunctional Tool for Genome-Wide Short Tandem Repeat Analysis for DNA, Transcripts, and Amino Acid Sequences with Integrated Primer Designer Click here for additional data file. Supplemental material, Supplementary_Figure1_revised_xyz152315147f517 for OSTRFPD: Multifunctional Tool for Genome-Wide Short Tandem Repeat Analysis for DNA, Transcripts, and Amino Acid Sequences with Integrated Primer Designer by Vivek Bhakta Mathema, Arjen M Dondorp and Mallika Imwong in Evolutionary Bioinformatics

## Supplemental Material

Supplementary_Figure2_revised_xyz15231acaf4c68 – Supplemental material for OSTRFPD: Multifunctional Tool for Genome-Wide Short Tandem Repeat Analysis for DNA, Transcripts, and Amino Acid Sequences with Integrated Primer Designer Click here for additional data file. Supplemental material, Supplementary_Figure2_revised_xyz15231acaf4c68 for OSTRFPD: Multifunctional Tool for Genome-Wide Short Tandem Repeat Analysis for DNA, Transcripts, and Amino Acid Sequences with Integrated Primer Designer by Vivek Bhakta Mathema, Arjen M Dondorp and Mallika Imwong in Evolutionary Bioinformatics

## Supplemental Material

Supplementary_Figure3_revised_xyz152312c689ebf – Supplemental material for OSTRFPD: Multifunctional Tool for Genome-Wide Short Tandem Repeat Analysis for DNA, Transcripts, and Amino Acid Sequences with Integrated Primer Designer Click here for additional data file. Supplemental material, Supplementary_Figure3_revised_xyz152312c689ebf for OSTRFPD: Multifunctional Tool for Genome-Wide Short Tandem Repeat Analysis for DNA, Transcripts, and Amino Acid Sequences with Integrated Primer Designer by Vivek Bhakta Mathema, Arjen M Dondorp and Mallika Imwong in Evolutionary Bioinformatics

## Supplemental Material

Supplementary_Figure4_revised_xyz15231d02c71b2 – Supplemental material for OSTRFPD: Multifunctional Tool for Genome-Wide Short Tandem Repeat Analysis for DNA, Transcripts, and Amino Acid Sequences with Integrated Primer Designer Click here for additional data file. Supplemental material, Supplementary_Figure4_revised_xyz15231d02c71b2 for OSTRFPD: Multifunctional Tool for Genome-Wide Short Tandem Repeat Analysis for DNA, Transcripts, and Amino Acid Sequences with Integrated Primer Designer by Vivek Bhakta Mathema, Arjen M Dondorp and Mallika Imwong in Evolutionary Bioinformatics

## Supplemental Material

Supplementary_Figure5_revised_xyz15231562c9f67 – Supplemental material for OSTRFPD: Multifunctional Tool for Genome-Wide Short Tandem Repeat Analysis for DNA, Transcripts, and Amino Acid Sequences with Integrated Primer Designer Click here for additional data file. Supplemental material, Supplementary_Figure5_revised_xyz15231562c9f67 for OSTRFPD: Multifunctional Tool for Genome-Wide Short Tandem Repeat Analysis for DNA, Transcripts, and Amino Acid Sequences with Integrated Primer Designer by Vivek Bhakta Mathema, Arjen M Dondorp and Mallika Imwong in Evolutionary Bioinformatics
