# Supplementary figures and images for: OSTRFPD: Multifunctional Tool for Genome-Wide Short Tandem Repeat Analysis for DNA, Transcripts, and Amino Acid Sequences with Integrated Primer Designer
Source: Evol Bioinform Online. 2019 Apr 23;15:1176934319843130. doi: 10.1177/1176934319843130 (PMC6482647; doi:10.1177/1176934319843130)

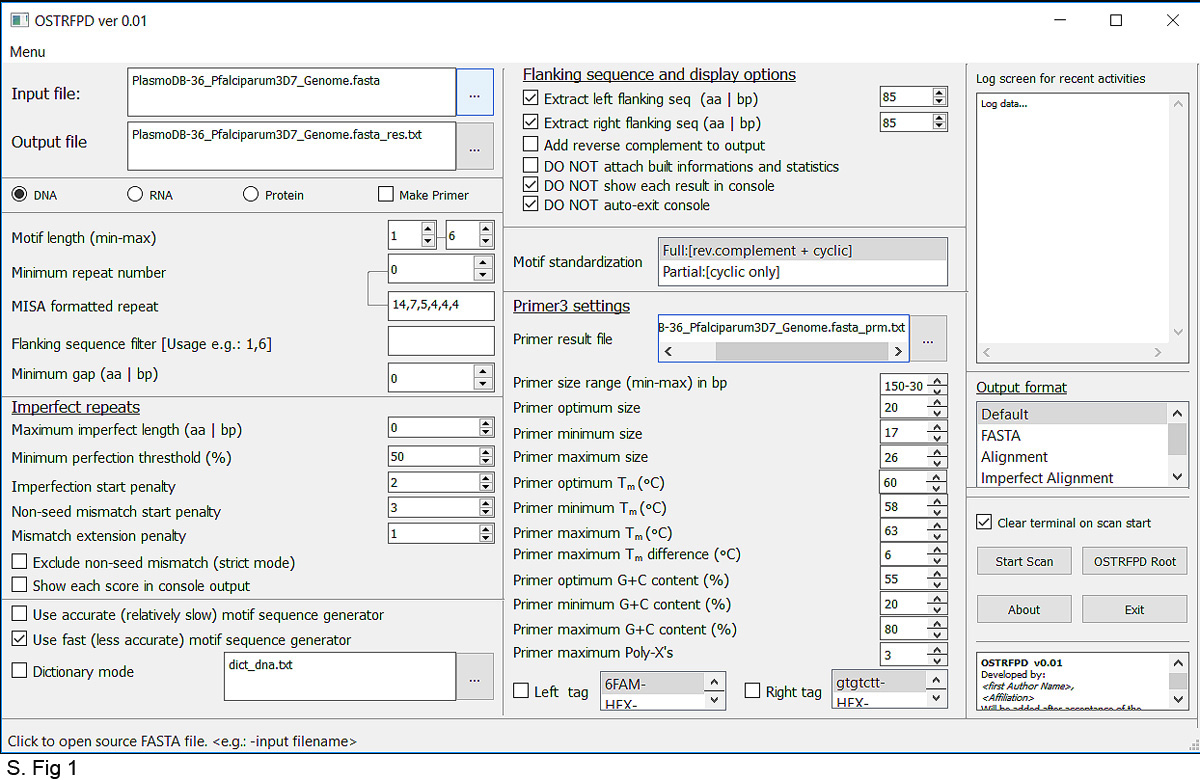

Supplement: Supplementary_Figure1_revised_xyz152315147f517 – Supplemental material for OSTRFPD: Multifunctional Tool for Genome-Wide Short Tandem Repeat Analysis for DNA, Transcripts, and Amino Acid Sequences with Integrated Primer Designer [file Supplementary_Figure1_revised_xyz152315147f517.jpg]

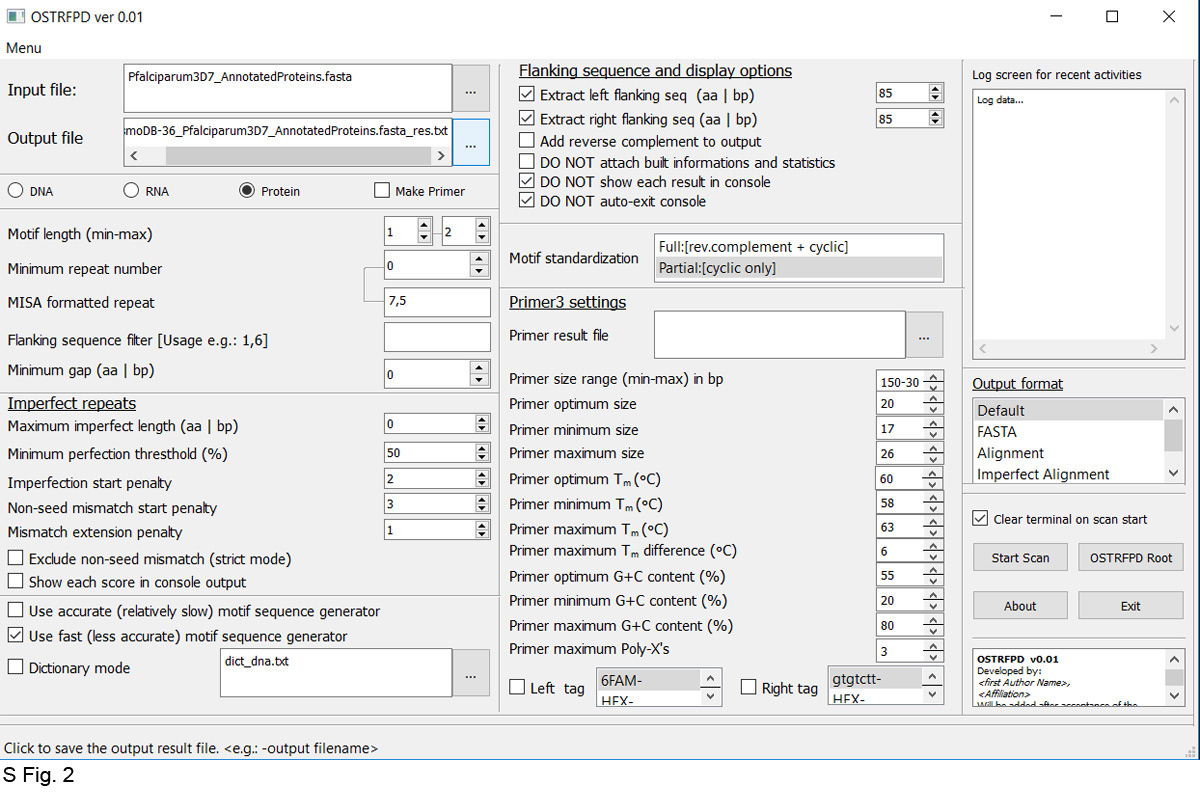

Supplement: Supplementary_Figure2_revised_xyz15231acaf4c68 – Supplemental material for OSTRFPD: Multifunctional Tool for Genome-Wide Short Tandem Repeat Analysis for DNA, Transcripts, and Amino Acid Sequences with Integrated Primer Designer [file Supplementary_Figure2_revised_xyz15231acaf4c68.jpg]

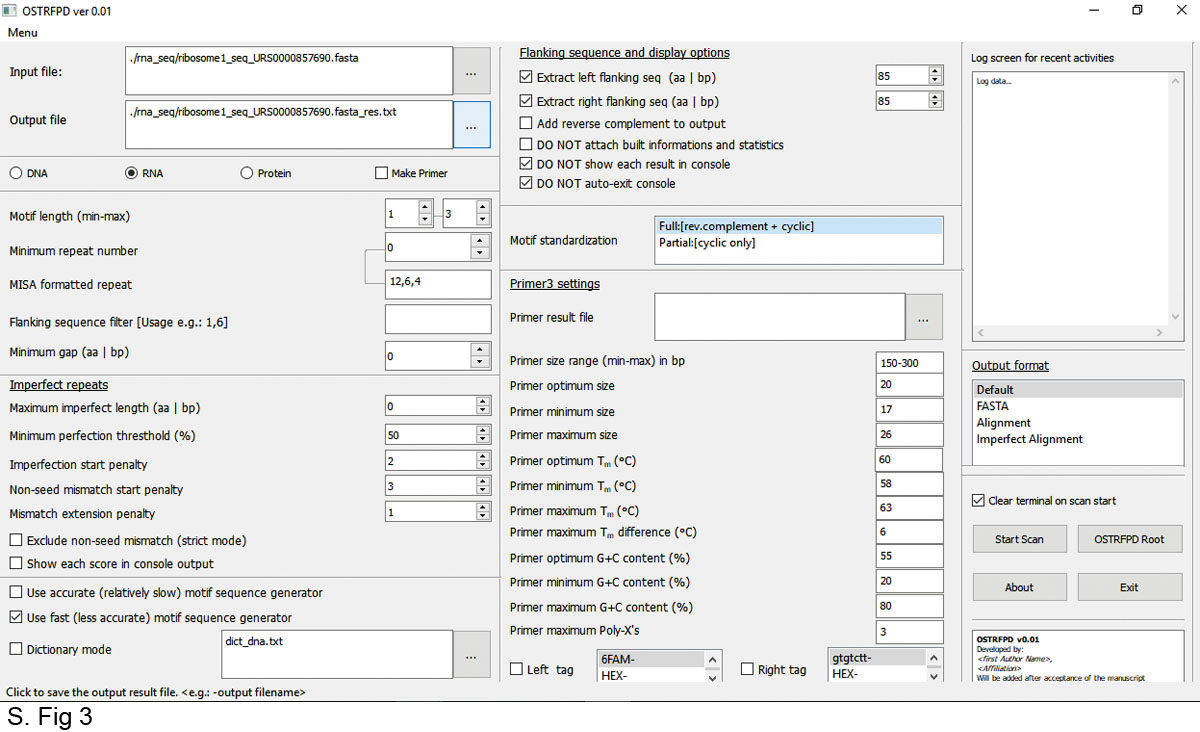

Supplement: Supplementary_Figure3_revised_xyz152312c689ebf – Supplemental material for OSTRFPD: Multifunctional Tool for Genome-Wide Short Tandem Repeat Analysis for DNA, Transcripts, and Amino Acid Sequences with Integrated Primer Designer [file Supplementary_Figure3_revised_xyz152312c689ebf.jpg]

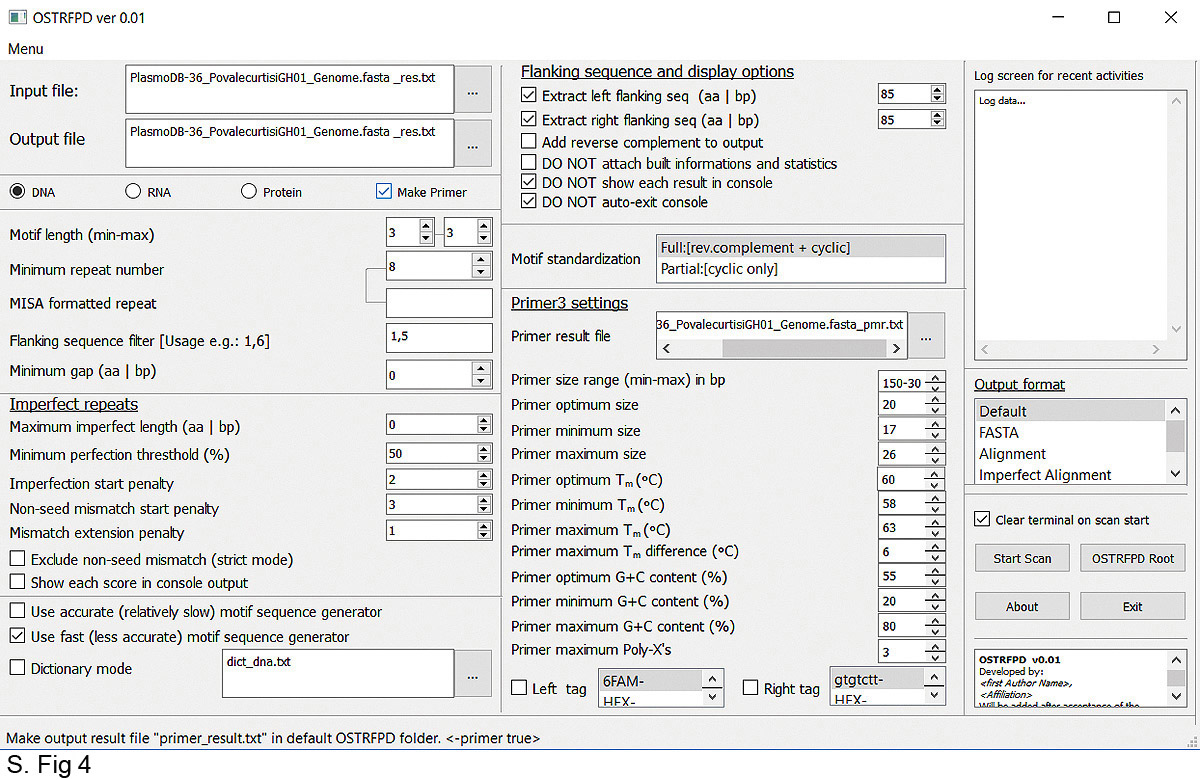

Supplement: Supplementary_Figure4_revised_xyz15231d02c71b2 – Supplemental material for OSTRFPD: Multifunctional Tool for Genome-Wide Short Tandem Repeat Analysis for DNA, Transcripts, and Amino Acid Sequences with Integrated Primer Designer [file Supplementary_Figure4_revised_xyz15231d02c71b2.jpg]

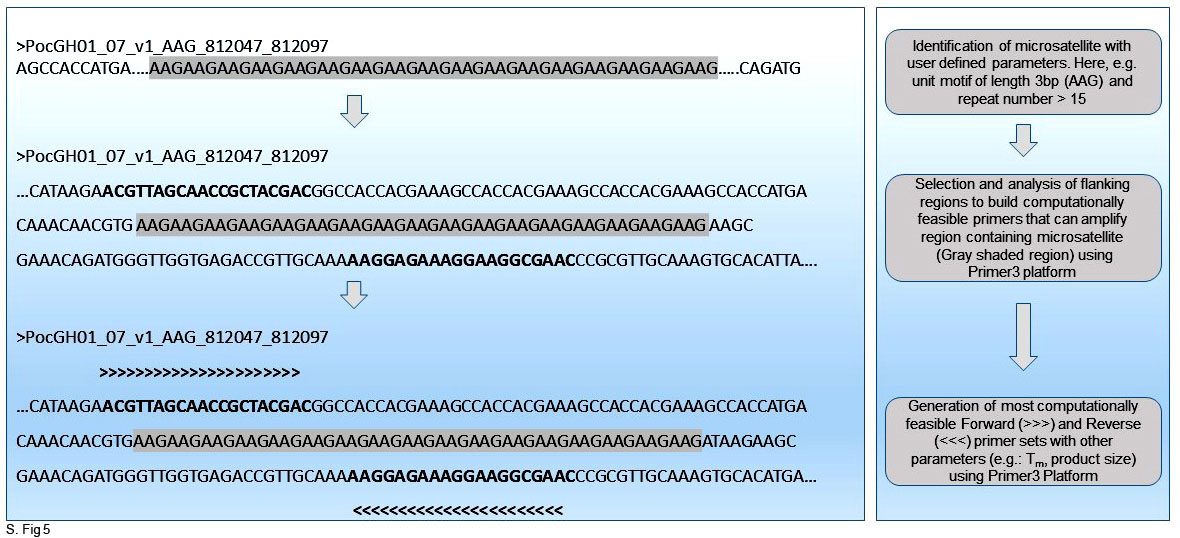

Supplement: Supplementary_Figure5_revised_xyz15231562c9f67 – Supplemental material for OSTRFPD: Multifunctional Tool for Genome-Wide Short Tandem Repeat Analysis for DNA, Transcripts, and Amino Acid Sequences with Integrated Primer Designer [file Supplementary_Figure5_revised_xyz15231562c9f67.jpg]
